# An Unusual Case of Iatrogenic Tracheal Diverticulum Found in a Mechanically Ventilated Patient: To Treat or Not to Treat

**DOI:** 10.7759/cureus.5911

**Published:** 2019-10-15

**Authors:** Munish Sharma, Chinthaka P Bulathsinghala, Alamgir Khan, Salim R Surani

**Affiliations:** 1 Internal Medicine, Corpus Christi Medical Center, Corpus Christi, USA; 2 Internal Medicine, University of North Texas, Dallas, USA; 3 Internal Medicine, Texas A&M Health Science Center, Temple, USA

**Keywords:** tracheal diverticulum, paratracheal air cyst, tracheocele, bronchoscopy, computed tomography chest

## Abstract

A tracheal diverticulum is an out pouching arising from the wall of the trachea, and its lumen is in communication with the tracheal lumen. It is a small air collection in the para-tracheal region and is an infrequently encountered clinical entity. This condition is often found incidentally on thoracic imaging and should be considered when para-tracheal air is present. It can be congenital or acquired. In the absence of symptoms, management is mainly conservative with close monitoring for complications such as para-tracheal abscess. We present a case of iatrogenic tracheal diverticulum that formed likely due to increased cuff pressure of the mispositioned endotracheal tube seated against a tracheal wall already vulnerable to injury due to multi-organ failure.

## Introduction

A tracheal diverticulum is a small air collection in the para-tracheal region. It has been clustered along with the tracheocele, lymphoepithelial cyst, and bronchogenic cyst as para-tracheal air cysts (PTACs) in the literature [1]. A tracheal diverticulum is an out pouching arising from the wall of the trachea, and its lumen is in communication with the tracheal lumen which is a major differentiating factor from tracheocele. The collective incidence of PTACs has been reported to range from 0.75% to 8.1% while the estimated incidence of tracheal diverticulum itself is around 2.4% only [2-3]. 

## Case presentation

A 34-year-old female with a past medical history significant for opioid abuse on methadone was brought to the emergency department (ED) with diffuse upper abdominal pain, nausea, and vomiting for three hours. She also complained of continuous vigorous bouts of productive cough for a week before admission. Her boyfriend, just before presentation, reportedly gave her an unknown dose of alprazolam, resulting in progressive lethargy and inability to maintain her airway. She was intubated with a 7.5 cm endotracheal tube (ET) in the ED.

**Figure 1:**
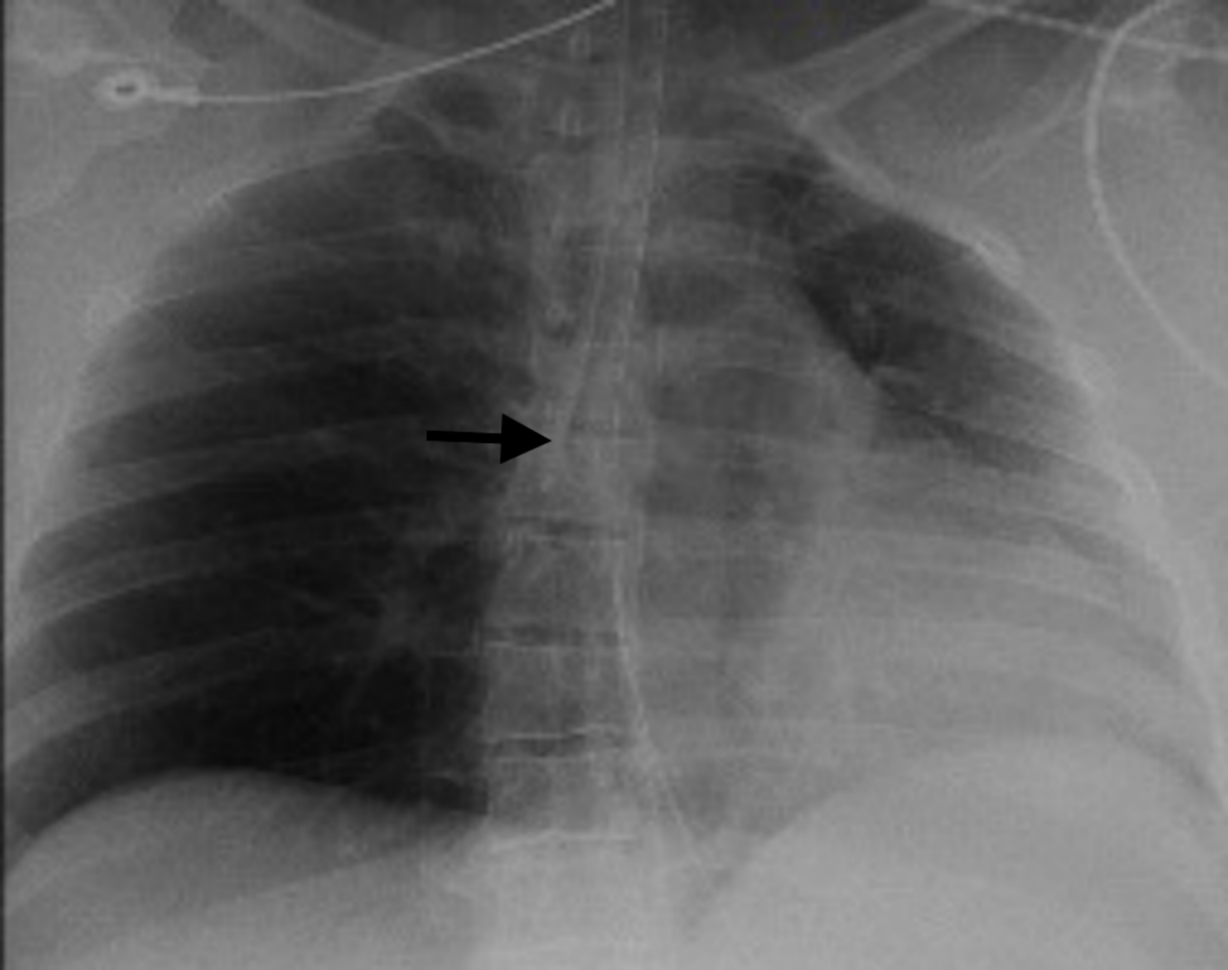
Chest X-ray showing endotracheal tube terminating at the level of carina directed towards the right main stem bronchus (black arrow)

**Figure 2:**
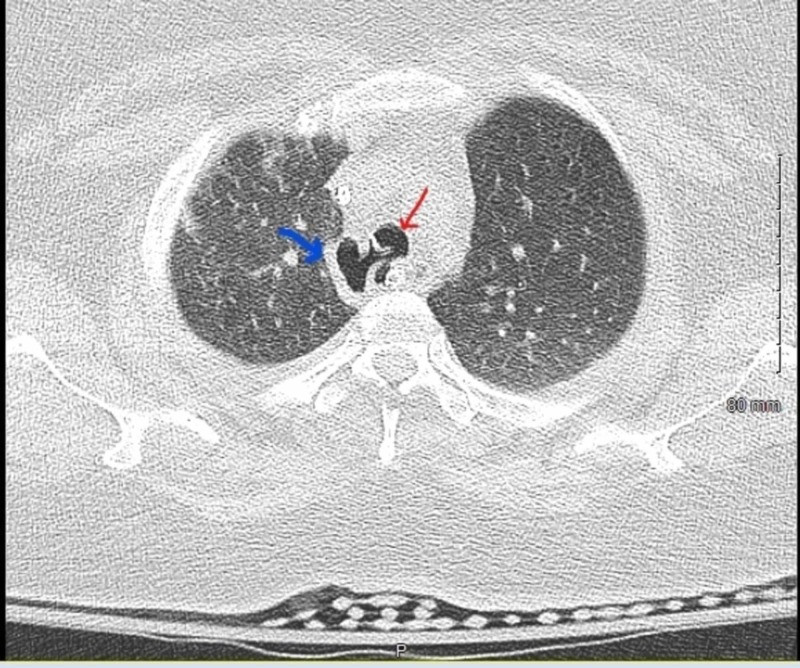
Computed tomography of the chest showing tracheal diverticulum at the right posterior lateral tracheal wall indicated by the blue arrow and trachea indicated by the red arrow

**Figure 3:**
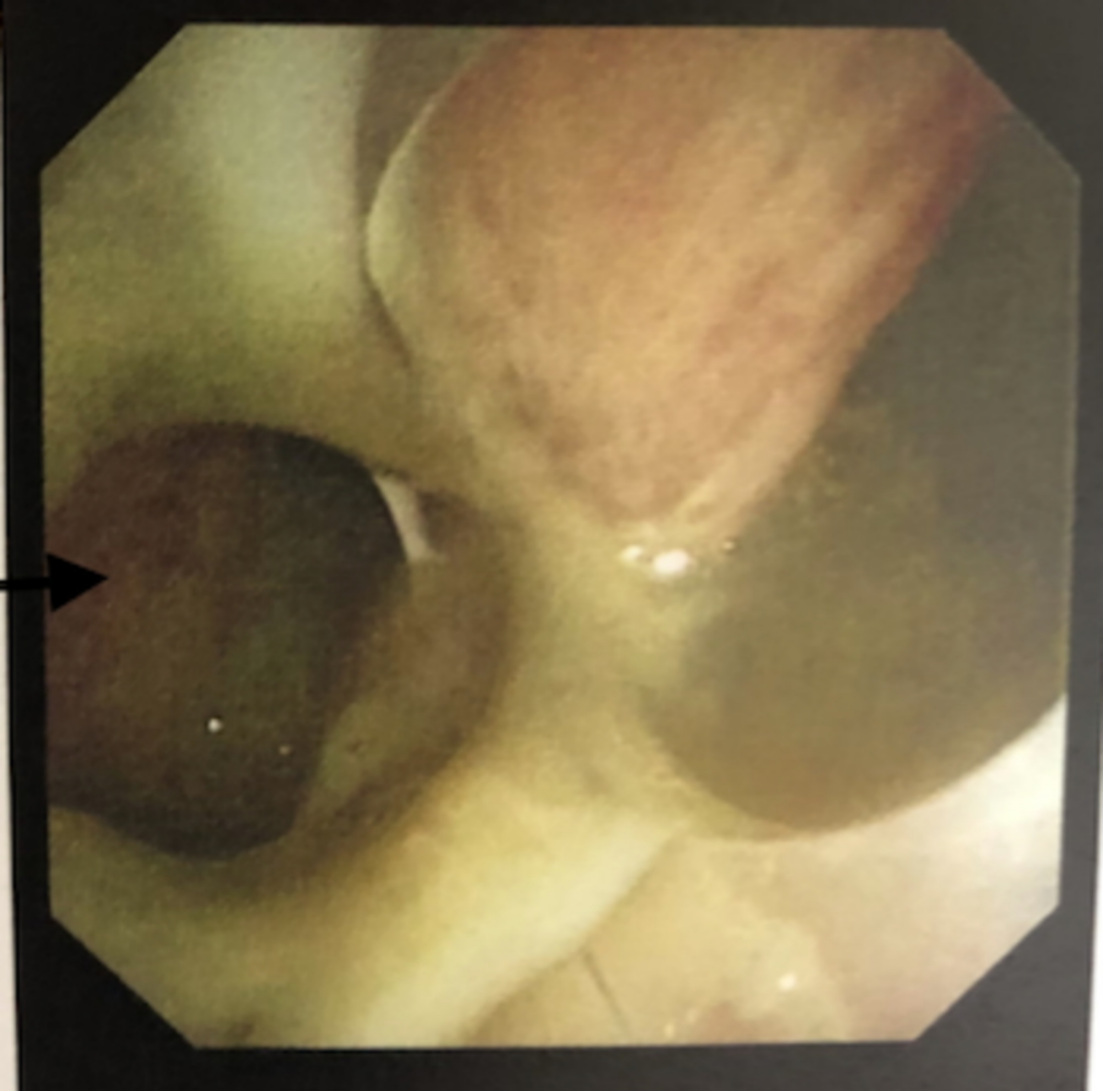
Bronchoscopy image showing defect at the right posterior lateral tracheal wall (black arrow)

The ET tube was found to be terminating at the level of the carina, directed towards the right main stem bronchus overnight in the ED (Figure 1). Once transferred to the intensive care unit, it was retracted to 3 cm above the carina after eight hours from intubation in the ED. Computed tomography (CT) of the chest without contrast showed a defect in the postern-lateral wall of the trachea with pneumo-mediastinum (Figure 2). Flexible bronchoscopy showed out pouching of approximately 2 x 4 cm base from the right postero-lateral aspect of the trachea (Figure 3). This tracheal out pouching was located around 0.5 cm above the carina.

**Figure 4:**
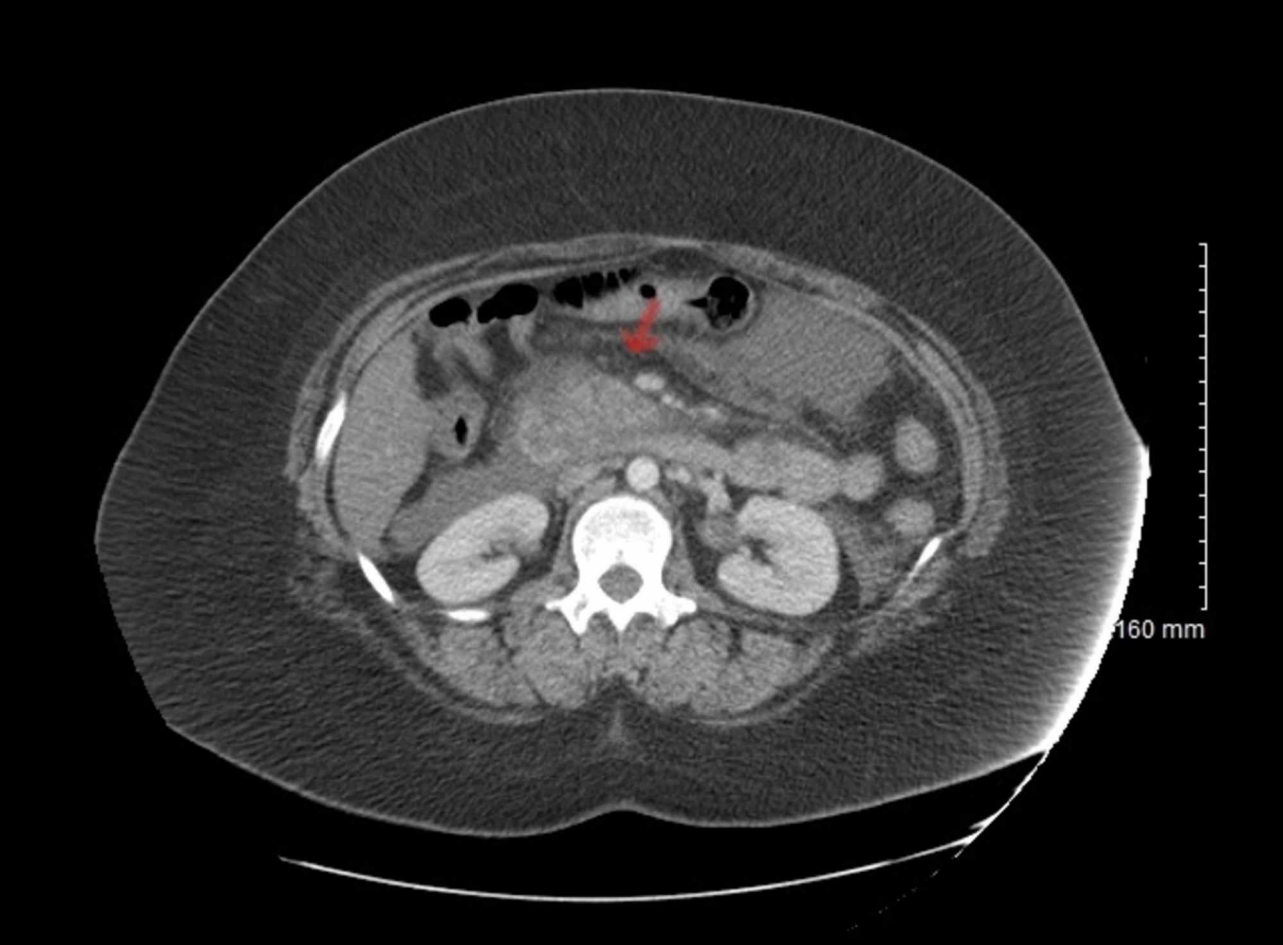
Computed tomography of the abdomen showed diffuse peri pancreatic inflammatory stranding and fluid (red arrow)

Bronchoscopy also revealed the copious thick yellowish secretions along with the sloughing of mucosa in the entire right bronchial tree. Inflammation was most pronounced in the right lower lobe. Broncho alveolar lavage was performed on the right lower lobe and culture samples grew methicillin-resistant *Staphylococcal aureus *(MRSA). She also had an elevated serum lipase level of 11,739 units/liter (reference range: 73-393). CT of the abdomen showed diffuse peripancreatic inflammatory stranding and fluid consistent with pancreatitis (Figure 4).

**Figure 5:**
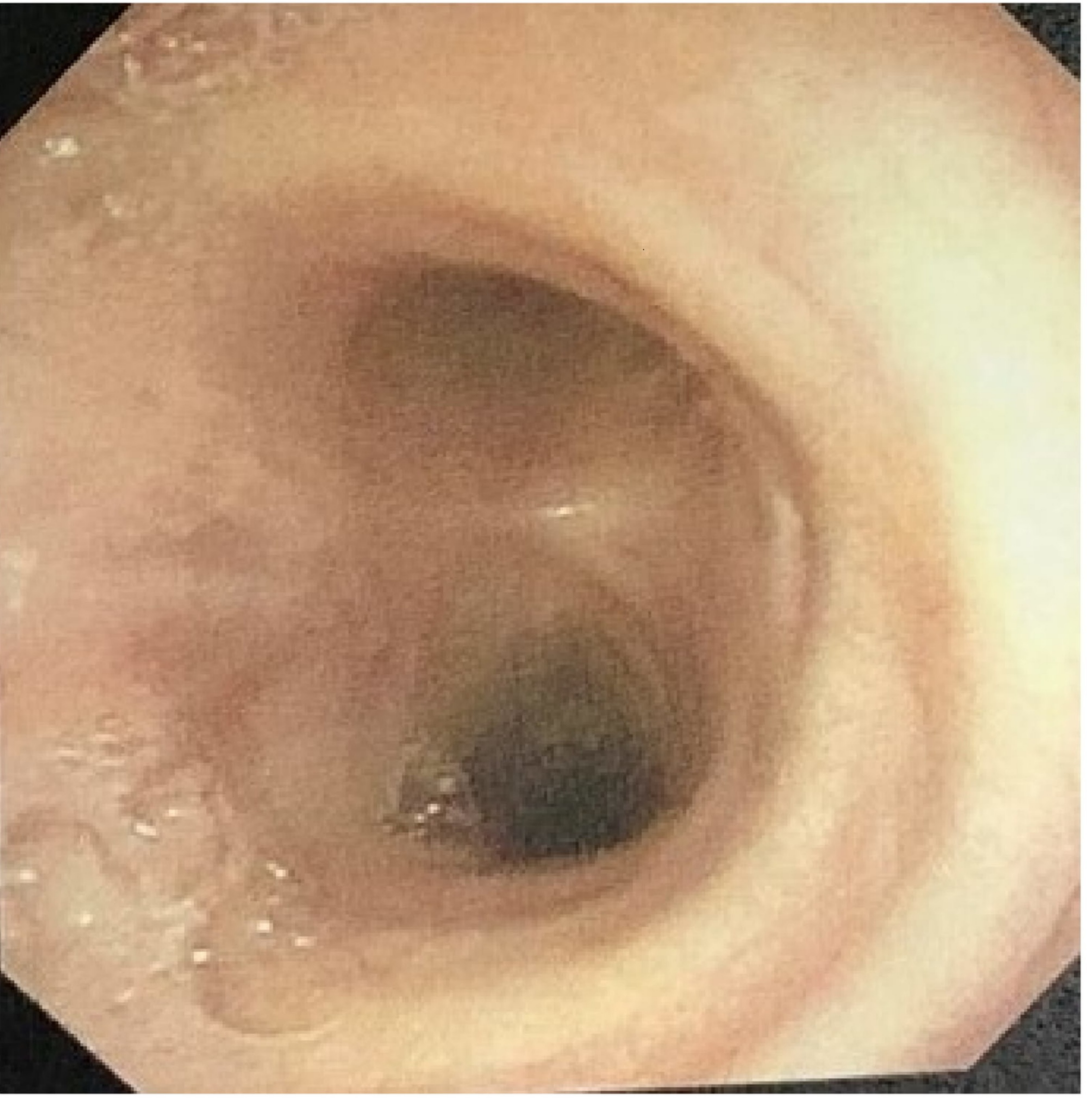
No evidence of tracheal defect in repeat bronchoscopy performed after a month

The patient's serum alcohol and triglyceride levels were within normal limits. She was kept intubated as she failed the weaning trial. She was treated with intravenous vancomycin for MRSA pneumonia. Thoracic surgery was consulted to consider surgical management of the tracheal diverticulum and was recommended against surgery with close observation for the development of abscess. A repeat CT chest three days post admission showed stable tracheal defect without evidence of perforation or abscess. The patient's lipase level trended down to 368 units/liter after five days. A repeat bronchoscopy did not reveal any change in the tracheal diverticulum. Connective tissue disease work-up, urine drug screen, and serology for hepatitis and human immunodeficiency virus (HIV) were negative. The patient was successfully extubated after five days of intubation. The patient was discharged home on room air with no respiratory complications. A repeat bronchoscopy after one month showed a complete resolution of the tracheal diverticulum (Figure 5).

## Discussion

A tracheal diverticulum is usually asymptomatic and an incidental finding on chest CT. It is mostly found in the right postern-lateral region of the trachea (97.1%) and rarely on the left side (2.9%) [3]. Tracheal diverticula can be congenital or acquired. Congenital diverticula are formed due to due to defective differentiation of the endodermal layer of the posterior membrane of the trachea [4]. Thus, it is a true diverticulum as smooth muscle and respiratory epithelium are all present in the out pouching. It is more common in males and often has a narrow opening that may be difficult to see, even with direct visualization by bronchoscopy [4]. In contrast, the acquired diverticulum is lined by respiratory epithelium only and is thus a pseudodiverticulum [5]. It is usually found at the level of the thoracic inlet, has a wider mouth and is larger compared to congenital diverticulum [1]. An acquired diverticulum can occur due to tracheomalacia or as a complication of thoracic surgeries. An increase in intraluminal tracheal pressure in patients with chronic cough coupled with the weakness of the tracheal wall due to repeated respiratory infections has also been reported to cause tracheal diverticulum [6]. In our case, incidental finding of tracheal diverticulum on the posterolateral wall of the trachea just above the carina was likely due to increased cuff pressure with spontaneous resolution within a month indicating it as an acquired diverticulum. Moreover, it had a large mouth with clear visibility during bronchoscopy which favors its acquired nature as discussed previously. We cannot exactly determine the reason for such a tracheal diverticulum formation in our patient; however, we postulated that critical illness due to acute pancreatitis and ensuing systemic inflammatory response syndrome along with MRSA pneumonia could have weakened and predisposed the posterior tracheal wall to form a pseudo diverticulum secondary to the mispositioned endotracheal tube and its cuff pressure. 

Multi detector CT (MDCT) with slice thickness < 1 mm is better for detection and more detail evaluation of tracheal diverticulum [7] as it may be difficult to assess a tracheal diverticulum especially with a very narrow opening with the use of bronchoscopy [6]. The major complication is the risk of secondary infection and the potential formation of a paratracheal abscess [8]. Our patient was treated with intravenous vancomycin for MRSA pneumonia and a repeat CT chest in three days was performed to ensure that a paratracheal abscess was not forming. In the absence of definitive management guidelines, especially in a critically ill patient on a ventilator, it was difficult to devise an accurate management plan. We also discussed the case with thoracic surgery that also recommended managing the case conservatively with close monitoring for the aforementioned complications as there is no evidence in the literature for preemptive surgical resection of the diverticulum [9]. Minimal positive expiratory pressure and lower tidal volume were used to avoid any volume-trauma and emphasized on early liberation from mechanical ventilation.

## Conclusions

Incidental findings of the paratracheal air sac in a patient should raise suspicion for tracheal diverticulum. Asymptomatic acquired cases should be managed conservatively with close surveillance for complications. Besides unknown etiology and chronicity, ongoing concomitant acute pancreatitis, MRSA pneumonia, and potential risk of worsening of the paratracheal air cyst due to mechanical ventilation, added to the complexity of our case. In the absence of established clinical guidelines for such a unique and complex case scenario, this patient was managed conservatively with close monitoring of the diverticulum with repeat imaging and interval bronchoscopy.
